# SARS-CoV-2 IgG seropositivity after the severe Omicron wave of COVID-19 in Hong Kong

**DOI:** 10.1080/22221751.2022.2106899

**Published:** 2022-09-05

**Authors:** Rosana Wing-Shan Poon, Brian Pui-Chun Chan, Wan-Mui Chan, Carol Ho-Yan Fong, Xiaojuan Zhang, Lu Lu, Lin-Lei Chen, Joy-Yan Lam, Vincent Chi-Chung Cheng, Samson S. Y. Wong, Kin-Hang Kok, Kwok-Yung Yuen, Kelvin Kai-Wang To

**Affiliations:** a Department of Microbiology, Queen Mary Hospital, Pokfulam, Hong Kong Special Administrative Region, People’s Republic of China; b State Key Laboratory for Emerging Infectious Diseases, Carol Yu Centre for Infection, Department of Microbiology, School of Clinical Medicine, Li Ka Shing Faculty of Medicine, The University of Hong Kong, Pokfulam, Hong Kong Special Administrative Region, People’s Republic of China; c Centre for Virology, Vaccinology and Therapeutics, Hong Kong Science and Technology Park, Pak Shek Kok, Hong Kong Special Administrative Region, People’s Republic of China

**Keywords:** COVID-19, SARS-CoV-2, serosurveillance, receptor binding domain, nucleoprotein, ORF8

## Abstract

The SARS-CoV-2 Omicron variant has led to a major wave of COVID-19 in Hong Kong between January and May 2022. Here, we used seroprevalence to estimate the combined incidence of vaccination and SARS-CoV-2 infection, including subclinical infection which were not diagnosed at the acute stage. The overall seropositive rate of IgG against receptor binding domain (anti-RBD IgG) increased from 52.2% in December 2021 to 89.3% in May 2022. The level of anti-RBD IgG was lowest in the 0–9 and ≥80 year-old age groups in May 2022. The seropositive rate of antibody against ORF8, which reflects the rate of prior infection, was 23.4% in May 2022. Our data suggest that although most individuals were either vaccinated or infected after the fifth wave, children and older adults remain most vulnerable. Public health measures should target these age groups in order to ameliorate the healthcare consequences of upcoming waves.

The severe acute respiratory syndrome coronavirus 2 (SARS-CoV-2) Omicron variant emerged in November 2021 [1,2]. The combination of high contagiousness [3], together with the ability to escape prior immunity [4,5], facilitated the Omicron variant to cause outbreaks even in areas employing an elimination strategy [6,7]. In Hong Kong, although BNT162b2 and CoronaVac vaccination began since February 2021, the number of COVID-19 cases confirmed by reverse transcription-polymerase chain reaction (RT–PCR) or antigen test increased rapidly between January and March 2022 during the Omicron-dominant fifth wave [6], and exceeded one million in March 2022.

Previous studies suggested that patients with either infection or vaccination have a lower risk of severe infection [8,9]. Therefore, knowledge on the proportion of the population who have been vaccinated or infected would be critical for risk assessment of upcoming waves. Although the number of patients with vaccination is well documented, the actual number of infected individuals is uncertain since many patients were not tested at the time of acute illness.

In order to attain a more accurate age-specific estimate of the proportion of the Hong Kong population who have been infected and protected, we conducted a cross-sectional seroprevalence study. We retrieved a total of 873 anonymized archived serum or plasma specimens collected in December 2021 and May 2022 in Hong Kong (Supplementary Table S1) [10]. Details of the blood specimens, antibody assays and statistical analysis are presented in the Supplementary Methods.

We first assessed the seropositive rate of immunoglobulin G against the SARS-CoV-2 RBD (anti-RBD IgG) [11]. Anti-RBD IgG can be elicited by prior infection or vaccination. The overall seropositive rate of anti-RBD IgG increased from 52.2% (221/423) in December 2021 to 89.3% (402/450) in May 2022 (*P* < 0.001). The greatest increase of anti-RBD IgG seropositive rates occurred in the 0–9 and ≥80 year-old age groups (absolute difference between May 2022 and December 2021: 70% for 0–9 year-old and 61% for ≥80 year-old) (Figure 1(A)). For both time periods, the anti-RBD IgG seropositive rate was highest in the 20–29 year-old age group (75% [39/52] in December 2021; 100% [50/50] in May 2022) but lowest in the 0–9 year-old age group (0% [0/19] in December 2021; 70% [35/50] in May 2022). The anti-RBD IgG seropositive rate of the 0–9 year-old age-group was significantly lower than those of other age groups (70% [35/50] vs 91.8% [367/400]; *P* < 0.0001) in May 2022. Within the 0–9 year-old age group, those aged 1–2 years had a significantly lower anti-RBD IgG seropositive rate than those aged 3–9 years old (33% [3/9] vs 79% [27/34]; *P* = 0.0137) in May 2022 (Figure 1(B)).

**Figure 1.**
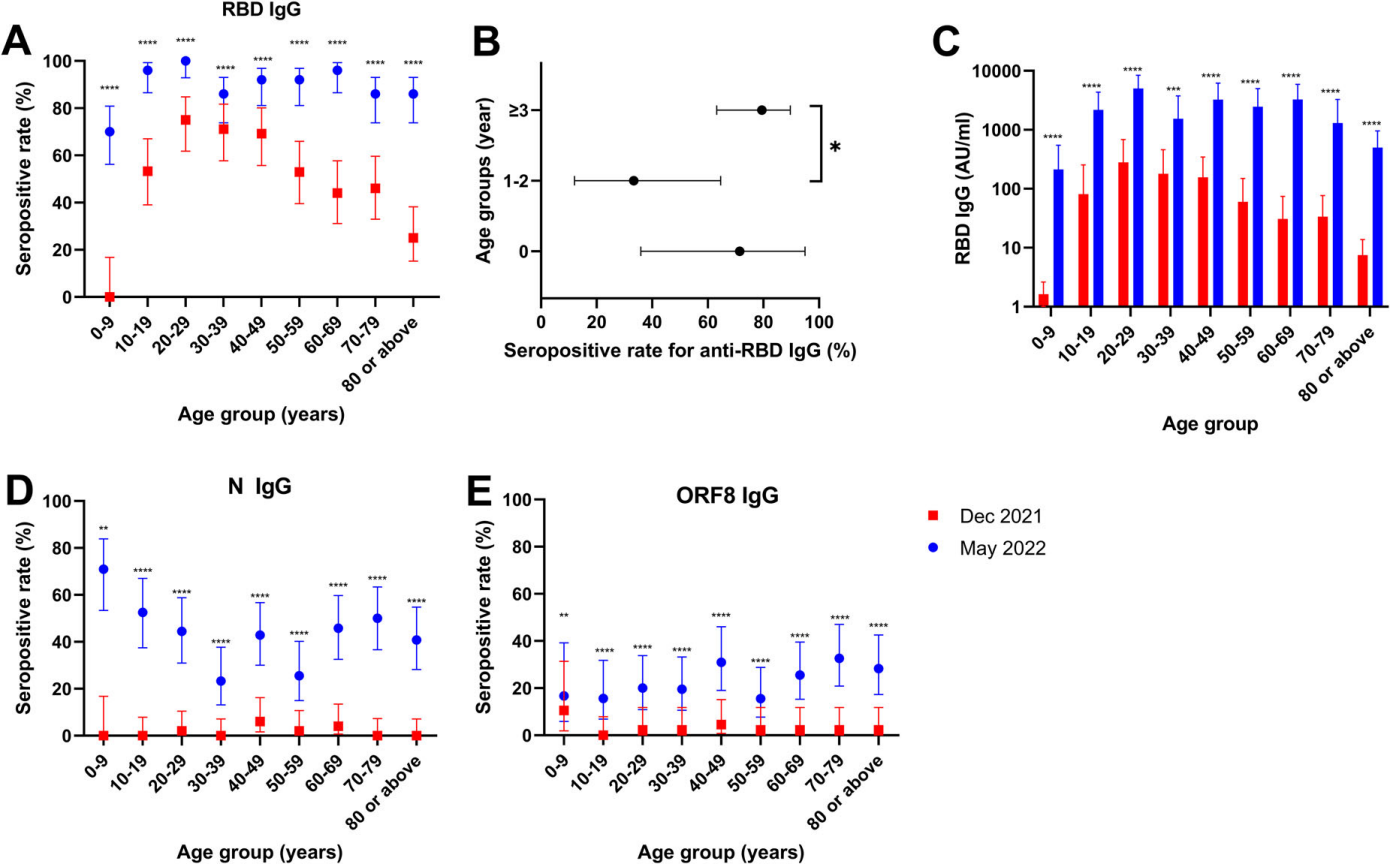
Serosurveillance of the Hong Kong population in December 2021 and May 2022. (A) Age-specific anti-RBD IgG seropositive rates. Data represent the seropositive rate, and the error bar represents the 95% confidence interval. (B) Comparison of seropositive rates among <1, 1–2 and 3–9 year-old individuals in May 2022. Data represent the seropositive rate, and the error bar represents the 95% confidence interval. (C) Age-specific anti-RBD IgG levels in December 2021 and May 2022. Data represent geometric mean and error bar represents the 95% confidence interval. (D&E) Age-specific anti-N (D) and anti-ORF8 (E) IgG seropositive rates. Data represent the seropositive rate, and the error bar represents the 95% confidence interval. *P < 0.05; **P < 0.01; ***P < 0.001; ****P < 0.0001.

We also analysed the level of anti-RBD IgG in our population. There was a significant increase in the geometric mean anti-RBD IgG level in all age groups (Figure 1(C)). In December 2021 and May 2022, the geometric mean anti-RBD IgG level was highest in the 20–29 year-old age group. In May 2022, the geometric mean anti-RBD IgG levels of the 0–9 year-old (213 AU/ml) and >80 year-old (500 AU/ml) age groups were 23.7- and 10.1-fold lower than that of the 20–29 year-old age group (5046 AU/ml). To further delineate the levels of anti-RBD IgG among vaccinated or infected patients, we retrieved serum specimens from patients infected with Omicron without prior vaccination (Omicron/non-vaccinated), those infected with Omicron with prior vaccination (breakthrough Omicron), and those who have received 2 doses of vaccination without infection (2-dose-vaccinated). The breakthrough Omicron group (median, 16184 AU/ml; interquartile range [IQR], 6104-27503) had significantly higher anti-RBD IgG level than the Omicron/non-vaccinated group (median, 360.1 AU/ml; IQR, 3.9-18372) or the 2-dose vaccinated group (median, 5830; IQR, 1076-12772) (*P* = 0.0108) (Supplementary Figure S1).

One drawback of anti-RBD IgG is that it cannot differentiate between infection and vaccination. Anti-N IgG has been used in estimating the prevalence of natural infection in areas without the use of inactivated virus vaccines [12]. However, the Hong Kong vaccination programme includes CoronaVac, an inactivated virus vaccine, which can elicit anti-N IgG. On the other hand, ORF8 is only produced during infection, and therefore anti-ORF8 IgG should only be present in patients with infection [13]. The overall seropositive rate of anti-N IgG increased from 1.7% (7/412) to 43.2% (172/398) (Figure 1(D)), while the seropositive rate for anti-ORF8 IgG increased from 2.7% (10/372) to 23.4% (86/367) (Figure 1(E)). The seropositive rates of anti-ORF8 IgG was slightly higher among older adults (>25% for those aged ≥60 years) than children and younger adults (≤20% for those aged <40 years).

## Discussion

Our data showed that there was a significant increase in the overall anti-RBD IgG seropositive rate in Hong Kong between December 2021 (just before the Omicron wave) and May 2022 (two months after the peak of the Omicron wave). The high overall seropositive rate (89.3%) was driven by the large number of infections and increased vaccination uptake rate during the Omicron wave. The results from our current serosurveillance concur with our previous estimates of protection, 72.9–88.7%, using data from vaccination and RT–PCR or antigen test-confirmed infection [14]. With this high seropositive rate, Hong Kong may be protected from a large outbreak of severe COVID-19 with antigenically similar strains. However, as SARS-CoV-2 continues to evolve, we should continue to monitor for novel variants which can escape our population immunity and cause future outbreaks.

In this study, we used an anti-RBD IgG assay to determine the seroprevalence. We previously showed that anti-RBD IgG was more reliable than anti-N IgG or neutralizing antibody test in documenting past infection [11]. Sauré *et al* showed that >90% of BNT162b2 vaccine recipients, but only 47.3% of CoronaVac recipients, were seropositive for anti-RBD IgG at 16 weeks after second dose vaccination [15]. Therefore, anti-RBD IgG is the most reliable serological marker for past infection or BNT162b2 vaccination, but may underestimate the number of individuals with CoronaVac vaccine. The overall anti-ORF8 IgG seropositive rate, which represent the rate of prior infection, was 23.4% in May 2022, which was 1.4-fold higher than the RT–PCR/antigen test positive rate (16.2% [1,204,210/7,413,070] as of April 30, 2022). Thus, our seroprevalence study also reveals the hidden burden of infection. A limitation in our study is that since we included archived specimens from patients attending the hospital, our seroprevalence data may not be representative of health individuals without comorbidities.

Our results suggested that although the overall seropositive rate was high in May 2022, the population aged 0–9 years and those aged ≥80 years old remain vulnerable. Within the 0–9 year-old age group, those aged 1–2 years old had a much lower seropositive rate than and those 3–9 years old (33% vs 79%). This is likely because the 1–2 year-old children could not benefit from maternal antibodies and were not eligible for COVID-19 vaccination. For older adults aged ≥80 years, although their seropositive rate was 86%, the geometric mean anti-RBD IgG titers were 10.1-fold lower than younger adults in May 2022. Public health measures should specifically target these age groups. Our data would also be useful to other places with similar public health strategies as in Hong Kong.

## Supplementary Material

Supplemental Material

## References

[1] Wong SC, Au AK, Chen H, et al. Transmission of Omicron (B.1.1.529) – SARS-CoV-2 variant of concern in a designated quarantine hotel for travelers: a challenge of elimination strategy of COVID-19. Lancet Reg Health West Pac. 2022;18:100360. 34961854 10.1016/j.lanwpc.2021.100360 PMC8696199

[2] Madhi SA, Kwatra G, Myers JE, et al. Population immunity and Covid-19 severity with Omicron variant in South Africa. N Engl J Med. 2022;386:1314–1326. 35196424 10.1056/NEJMoa2119658 PMC8908853

[3] Cheng VC, Fung KS, Siu GK, et al. Nosocomial outbreak of COVID-19 by possible airborne transmission leading to a superspreading event. Clin Infect Dis. 2021;73:e1356–e1364. 33851214 10.1093/cid/ciab313 PMC8083289

[4] Lu L, Mok BW, Chen LL, et al. Neutralization of SARS-CoV-2 Omicron variant by sera from BNT162b2 or coronavac vaccine recipients. Clin Infect Dis. 2021. doi:10.1093/cid/ciab1041:ciab1041. PMC8754807 34915551

[5] Chen LL, Chua GT, Lu L, et al. Omicron variant susceptibility to neutralizing antibodies induced in children by natural SARS-CoV-2 infection or COVID-19 vaccine. Emerg Microbes Infect. 2022: 1–17. doi:10.1080/22221751.2022.2035195. PMC8843159 35084295

[6] Cheng VC, Ip JD, Chu AW, et al. Rapid spread of SARS-CoV-2 Omicron subvariant BA.2 in a single-source community outbreak. Clin Infect Dis. 2022. doi:10.1093/cid/ciac203:ciac203. PMC8992238 35271728

[7] Cai J, Deng X, Yang J, et al. Modeling transmission of SARS-CoV-2 Omicron in China. Nat Med. 2022;28:1468–1475. 35537471 10.1038/s41591-022-01855-7 PMC9307473

[8] Altarawneh HN, Chemaitelly H, Hasan MR, et al. Protection against the Omicron variant from previous SARS-CoV-2 infection. N Engl J Med. 2022;386:1288–1290. 35139269 10.1056/NEJMc2200133 PMC8849180

[9] Gray G, Collie S, Goga A, et al. Effectiveness of Ad26.COV2.S and BNT162b2 vaccines against Omicron variant in South Africa. N Engl J Med. 2022;386:2243–2245. 35507482 10.1056/NEJMc2202061 PMC9093716

[10] Chen LL, Abdullah SM, Chan WM, et al. Contribution of low population immunity to the severe Omicron BA.2 outbreak in Hong Kong. Nat Commun. 2022;13:3618. 10.1038/s41467-022-31395-0 PMC9232516 35750868

[11] Lu L, Chen LL, Zhang RR, et al. Boosting of serum neutralizing activity against the Omicron variant among recovered COVID-19 patients by BNT162b2 and CoronaVac vaccines. EBioMedicine. 2022;79:103986. 35398786 10.1016/j.ebiom.2022.103986 PMC8989491

[12] Clarke KEN, Jones JM, Deng Y, et al. Seroprevalence of infection-induced SARS-CoV-2 antibodies – United States, September 2021–February 2022. Morb Mortal Wkly Rep. 2022;71:606–608. 10.15585/mmwr.mm7117e3 PMC9098232 35482574

[13] Wang X, Lam JY, Wong WM, et al. Accurate diagnosis of COVID-19 by a novel immunogenic secreted SARS-CoV-2 orf8 protein. mBio. 2020: 11: e02431-20. 10.1128/mBio.02431-20 PMC7587431 33082264

[14] Lung DC, Sridhar S, Yuen KY. 高築屏障護民康 復常無懼浪接浪. Ming Pao. [cited 2022 May 25]. Available from: https://mmingpaocom/pns/%e8%a7%80%e9%bb%9e/article/20220520/s00012/1652984046769/%e9%be%8d%e6%8c%af%e9%82%a6-%e8%96%9b%e9%81%94-%e8%a2%81%e5%9c%8b%e5%8b%87-%e9%ab%98%e7%af%89%e5%b1%8f%e9%9a%9c%e8%ad%b7%e6%b0%91%e5%ba%b7-%e5%be%a9%e5%b8%b8%e7%84%a1%e6%87%bc%e6%b5%aa%e6%8e%a5%e6%b5%aa.

[15] Saure D, O'Ryan M, Torres JP, et al. Dynamic IgG seropositivity after rollout of CoronaVac and BNT162b2 COVID-19 vaccines in Chile: a sentinel surveillance study. Lancet Infect Dis. 2022;22:56–63. 34509185 10.1016/S1473-3099(21)00479-5 PMC8428469

