# Supplementary material for: SARS-CoV-2 IgG seropositivity after the severe Omicron wave of COVID-19 in Hong Kong
Source: Emerg Microbes Infect. 2022 Sep 5;11(1):2116–9. doi: 10.1080/22221751.2022.2106899 (PMC9448364; doi:10.1080/22221751.2022.2106899)
Supplement: Supplemental Material [file TEMI_A_2106899_SM7300.docx]

**SUPPLEMENTARY METHODS**

**Patients**

Anonymised archived plasma specimens from the clinical biochemistry laboratory of Queen Mary Hospital in Hong Kong were retrieved. The archived specimens were collected in December 2021 and May 2022, and encompassed all age groups from 0-9 years old to ≥80 years old. The number of specimens in each age group for anti-RBD IgG, anti-N IgG and anti-ORF8 IgG is shown in Supplementary Table S1. Blood specimens were excluded if the volume was insufficient for anti-RBD IgG testing. We also retrieved serum from patients infected with Omicron without prior vaccination (Omicron/non-vaccinated), those infected with Omicron with prior vaccination (breakthrough Omicron), and those who have received 2 doses of vaccination without infection (2-dose-vaccinated). This study was approved by the Institutional Review Board of the University of Hong Kong/Hospital Authority Hong Kong West Cluster (UW 18–141 and UW-21-313). Written informed consent was waived since archived anonymized specimens were used.

**Antibody assays**

IgG against the SARS-CoV-2 RBD was measured using the SARS-CoV-2 IgG II Quant assay (Abbott Diagnostics, Chicago, USA). Specimens with an anti-RBD IgG level of ≥50 AU/ml, the manufacturer’s cutoff, was considered as seropositive. IgG against the SARS-CoV-2 N protein was measured using the SARS-CoV-2 IgG (Abbott Ireland Diagnostics Division, Ireland). Specimens with an anti-N IgG level of ≥1.4 AU/ml, the manufacturer’s cutoff, was considered as seropositive.

IgG against the ORF8 protein was measured using an enzyme immunoassay as we described previously [1,2]. To determine the cutoff value for positivity, we first calculated the mean optical density (OD) values and standard deviation (SD) of 94 archived anonymous serum samples from 2019, and the cutoff OD value for a positive result was set as the mean OD value plus 3 SD.

**Statistical analysis**

All statistical analysis was performed using SPSS 26.0 (IBM SPSS Statistics) or GraphPad Prism 9.3.1.471 for Windows (GraphPad Software, San Diego CA, USA). The seropositive rates between time periods were compared using McNemar test. Log-transformed anti-RBD IgG level was compared using Kruskal Wallis with Dunn’s multiple comparisons test. For the purpose of statistical analysis of the geometric mean, an anti-RBD IgG level of 0 is considered as 1 AU/ml; and a value of >40,000 AU/ml) (the maximum detection limit of the assay) is considered as 40,001 AU/ml. A *P* value of <0.05 was considered to be statistically significant.

**REFERENCES**

[1] Wang X, Lam JY, Wong WM, Yuen CK, Cai JP, Au SW, et al. Accurate Diagnosis of COVID-19 by a Novel Immunogenic Secreted SARS-CoV-2 orf8 Protein. mBio 2020;11

[2] Wang X, Lam JY, Chen L, Au SW, To KKW, Yuen KY, et al. Mining of linear B cell epitopes of SARS-CoV-2 ORF8 protein from COVID-19 patients. Emerg Microbes Infect 2021;10:1016-23

**SUPPLEMENTARY TABLE S1.** Number of specimens in each age group for anti-RBD, anti-N and anti-ORF8 IgG assay.

| **Age group** | **December 2021** | | | | **May 2022** | | | |
| --- | --- | --- | --- | --- | --- | --- | --- | --- |
|  | Anti-RBD | Anti-N^a^ | Anti-ORF8^a^ | Anti-RBD | | Anti-N^a^ | Anti-ORF8^a^ |  |
| 0-9 | 19 | 19 | 19 | 50 | | 31 | 18 |  |
| 10-19 | 45 | 45 | 45 | 50 | | 40 | 32 |  |
| 20-29 | 52 | 50 | 44 | 50 | | 45 | 45 |  |
| 30-39 | 52 | 50 | 44 | 50 | | 43 | 46 |  |
| 40-49 | 52 | 50 | 44 | 50 | | 49 | 42 |  |
| 50-59 | 51 | 49 | 44 | 50 | | 43 | 45 |  |
| 60-69 | 50 | 50 | 44 | 50 | | 48 | 47 |  |
| 70-79 | 50 | 49 | 44 | 50 | | 50 | 46 |  |
| >=80 | 52 | 50 | 44 | 50 | | 49 | 46 |  |
| Total | 423 | 412 | 372 | 450 | | 398 | 367 |  |

^a^ Some serum specimens were excluded due to insufficient volume.

**SUPPLEMENTARY FIGURE S1.** Comparison of anti-RBD IgG levels between patients infected with Omicron without prior vaccination (Omicron/non-vaccinated), those infected with Omicron with prior vaccination (breakthrough Omicron), and those who have received 2 doses of vaccination without infection (2-dose-vaccinated).
